# Studies on the *Stenothemusharmandi* species-group (Coleoptera, Cantharidae), with descriptions of two new species from China

**DOI:** 10.3897/BDJ.9.e68659

**Published:** 2021-08-25

**Authors:** Shujuan Ge, Xingke Yang, Haoyu Liu, Yuxia Yang

**Affiliations:** 1 The Key Laboratory of Zoological Systematics and Application, School of Life Science, Institute of Life Science and Green Development, Hebei University, Baoding, China The Key Laboratory of Zoological Systematics and Application, School of Life Science, Institute of Life Science and Green Development, Hebei University Baoding China; 2 Key Laboratory of Zoological Systematics and Evolution, Institute of Zoology, Chinese Academy of Sciences, Beijing, China Key Laboratory of Zoological Systematics and Evolution, Institute of Zoology, Chinese Academy of Sciences Beijing China

**Keywords:** *Stenothemusharmandi* species-group, female reproductive system, new species, China

## Abstract

**Background:**

The *Stenothemusharmandi* species-group has 10 species at present. They are *S.harmandi* (Bourgeois, 1902) (located in N. India, Nepal); *S.holosericus* Švihla, 2005, *S.orbiculatus* Švihla, 2005 and *S.subnitidus* Švihla, 2005 (N. India); *S.distortirudis* Y. Yang & X. Yang, 2014, *S.laticollis* Y. Yang & X. Yang, 2014, *S.parallelus* Y. Yang & X. Yang, 2014 and *S.septimus* Y. Yang & X. Yang, 2014 (China: Xizang); *S.fugongensis* Y. Yang & X. Yang, 2014 (China: Yunnan) and *S.leishanensis* Y. Yang & X. Yang, 2014 (China: Guizhou). In the present study, two previously-known species are classified into this species-group, *S.dentatus* Wittmer, 1974 and *S.alexandrae* Švihla, 2004, of which the latter as a subspecies of the former is upgraded to the specific level and another two new species are discovered and described.

**New information:**

Two new species of the *Stenothemusharmandi* species-group are described, *S.acuticollis* **sp. n.** (China: Yunnan) and *S.nigricolor* **sp. n**. (China: Xizang), which are illustrated with habitus photos and aedeagi of males, abdominal sternites VIII and internal genitalia of females. *S.alexandrae* Švihla, 2004 **stat. n.** is upgraded from a subspecies of *S.dentatus* Wittmer, 1974 and the two species are classified into this species-group. Characters of the female reproductive system are described for the first time for the following species: *S.distortirudis* Y. Yang & X. Yang, 2014; *S.laticollis* Y. Yang & X. Yang, 2014; *S.leishanensis* Y. Yang & X. Yang, 2014; *S.orbiculatus* Švihla, 2005; *S.septimus* Y. Yang & X. Yang, 2014 and *S.subnitidus* Švihla, 2005. Meanwhile, some additional distribution information is added for previously-described species. A key for the identification of all species is updated.

## Introduction

The genus *Stenothemus* Bourgeois, 1907 is a moderately diverse group in Cantharidae, which contains 75 species hitherto known in total (Wittmer 1974, Okushima and Satô 1997, Okushima and Satô 1999, Švihla 2004,Švihla 2005,Švihla 2011,Hsiao 2015, Hsiao et al. 2016, Yang et al. 2014, Yang et al. 2021a). *Stenothemus* can be distinguished from all other genera of Cantharinae by its simple tarsal claws in both sexes, the head with a pair of smooth impressions behind antennal fossae, pronotum with widely rounded anterior angles and laterally projecting posterior angles or quadrate with rectangular posterior angles (Švihla 2004), the fused parameres of aedeagus deeply cleft on the ventral side and the ventral process and dorsal plate of each paramere converging (Okushima and Satô 1999). Within the genus, only one species group, the *S.harmandi* species-group, is recognised.

The *S.harmandi* species-group was proposed by Švihla (2005) and, later, was reviewed by Yang et al. (2014). There are 10 species included in this group (Švihla 2005, Yang et al. 2014), mostly distributed in the Himalayan areas with some spreading to the Yunnan-Guizhou Plateau (Yang et al. 2014). The members of this species-group are diagnosed by the distinctive aedeagus, with both ventral process and dorsal plate of each paramere bent ventrad , the ventral process thickened apically in varying degrees in lateral view and the dorsal plate greatly narrowed apically in dorsal view (Švihla 2005); the abdominal tergite VIII of female is curled ventrad to enfold the sides of sternite VIII, which is strongly narrowed posterad (Yang et al. 2014).

In the present study, two new species of the *S.harmandi* species-group were discovered from Yunnan and Xizang, China and described under the names of *S.acuticollis* **sp. n.** and *S.nigricolor* **sp. n**. Meanwhile, two previously-described species are added into this group, *S.dentatus* Wittmer, 1974 and *S.dentatusalexandrae* Švihla, 2004, whose females were either unknown or neglected in the original descriptions. Furthermore, it is suggested that *S.dentatusalexandrae* Švihla, 2004 be upgraded from the subspecific level to an independent species, on the basis of examination of the types. Additionally, the reproductive system of the female for cantharid beetles has been shown to be useful in delimitation of the species-group (Okushima 2005) or *Stenothemus* species (Yang et al. 2021a), but it has remained unknown for the *S.harmandi* species-group until now; herein, we present the characters of this structure for the members of this group.

## Materials and methods

The studied material is deposited in the Institute of Zoology, Chinese Academy of Sciences, Beijing, China (IZAS), the Museum of Hebei University, Baoding, China (MHBU), the Naturhistorisches Museum Basel, Switzerland (NHMB) and the National Museum, Prague, Czech Republic (NMPC).

Genitalia of both sexes and abdominal sternites VIII of females were dissected and cleared in a solution of 10% sodium hydroxide (NaOH) and female genitalia were dyed with haematoxylin. In studying the morphology of the aedeagus or female genitalia, at least one specimen was dissected per species, more if any damage occurred during dissection. If the species had a rather wide distribution range, one specimen was dissected from each locality. The measurements were carried out with the aid of a Leica M205A stereomicroscope. Habitus photos were taken using a Leica M205A stereomicroscope and multiple layers were stacked using Combine ZM (Helicon Focus 5.3). Line drawings were made using a camera lucida attached to a Nikon SMZ1500 stereomicroscope, then edited in CorelDRAW 12 and Adobe Photoshop CS3.10.0.1.

Complete label data in Chinese were transliterated for type specimens. Body length was measured from the anterior margin of the clypeus to the elytral apex and body width across the humeral part of elytra. Morphological terminology of aedeagus follows that of Okushima (2005) and morphological terminology of female genitalia follows that of Brancucci (1980).

## Data resources

The information of the specimens in this paper is from the preserved specimens of IZAS and MHBU, as well as the type specimens of NMPC and NHMB, examined in this study.

## Taxon treatments

### Stenothemus harmandi

species-group

F46D0560-4B51-5536-9949-F0E6960D123E

#### Diagnosis

Body is usually brown and mixed with irregular dark brown markings, except for only a few which are uniformly dark brown or black. The posterior angles of pronotum are sharply protruding laterad or obtusely rounded. The aedeagus with both ventral process and dorsal plate of each paramere are bent ventrad, the ventral process is thickened apically in varying degrees in lateral view and the dorsal plate is greatly narrowed apically in dorsal view. In the female, the abdominal tergite VIII is curled ventrad to enfold lateral sides of sternite VIII, which is strongly narrowed posteriorly.

#### Distribution

China, India, Nepal.

### Stenothemus alexandrae

Švihla, 2004 stat. n.

4DF6392D-FB89-558C-A104-3450ED95A836

Stenothemus dentatus alexandrae Švihla, 2004: 196, figs. 142‒144.

#### Materials

- **Type status:**Holotype. **Occurrence:** recordedBy: Jan Schneider; individualCount: 1; sex: male; lifeStage: adult; **Taxon:** scientificName: *Stenothemusalexandrae*; **Location:** country: India; stateProvince: Sikkim; locality: Gantok env.; verbatimElevation: 2000‒2500 m; locationRemarks: Fambong-Lho forest; **Event:** eventDate: 8.‒15.07.1997; **Record Level:** language: en; collectionCode: Insects; ownerInstitutionCode: NMPC; basisOfRecord: Preserved Specimen

#### Distribution

India.

#### Notes

In the original publication (Švihla 2004), *S.alexandrae* was treated as a subspecies of *S.dentatus* Wittmer, 1974. However, differences between the two taxa have been found not only in the external appearance, but also in the structure of the aedeagus. In *S.alexandrae*, the body is uniformly dark brown, pronotum bears projecting and sharp posterior angles (Fig. 1a) and the dorsal plates of the aedeagus are three times as long as wide and separated from each other in dorsal view (Švihla 2004: figs. 142‒144). In comparison, *S.dentatus* has a pale yellow body, mixed with dark brown markings on the disc of the pronotum, elytra and legs (Fig. 1b) and the dorsal plates are 1.5 times as long as wide, converging in dorsal view (Švihla 2004: fig. 145). As these differences are sufficient to support their independent status, we suggest that *S.alexandrae* be recognised at the specific level.

Compared with others, the aedeagus of this species is distinctive from all other species, where the ventral process of each paramere is bent ventrad to a less extent, at an angle of 30 degrees to the median lobe. Probably for this reason, it was not included in the *S.harmandi* species-group by Švihla (2005), when he established this group. Here, it is included in this species-group, based on the structure of the aedeagus, of which both ventral process and dorsal plate of each paramere bent ventrad, although to a lesser extent, ventral process thickened terminally and dorsal plate narrowed apically. All these characteristics match the definition of *S.harmandi* species-group well (Švihla 2005), so *S.alexandrae* is suggested to be a member of this group.

### Stenothemus dentatus

Wittmer, 1974

E5BD790C-B8FC-518F-AAD5-CFE32EDEA5E1

Stenothemus dentatus Wittmer, 1974: 52, fig. 5.

#### Materials

- **Type status:**Paratype. **Occurrence:** recordedBy: F. Schmid; individualCount: 1; sex: female; lifeStage: adult; **Taxon:** scientificName: *Stenothemusdentatus*; **Location:** country: India; stateProvince: Assam; locality: Kameng, Bomdi La; verbatimElevation: 8800ft; **Event:** eventDate: 17.06.1961; **Record Level:** language: en; collectionCode: Insects; ownerInstitutionCode: NHMB; basisOfRecord: Preserved Specimen

#### Distribution

India.

#### Notes

*S.alexandrae* was treated as a subspecies of *S.dentatus* due to the similarity of aedeagus in the orginal description (Švihla 2004), suggesting their close relationship. For the same reason with *S.alexandrae*, *S.dentatus* was not included in the *S.harmandi* species-group by Švihla (2005). As what is noted for *S.alexandrae*, except for the ventral process bent ventrad to a lesser extent, other characteristics of the aedeagus of *S.dentatus* match the diagnosis of *S.harmandi* species-group well, including both ventral process and dorsal plate of each paramere bent ventrad, ventral process thickened terminally and dorsal plate narrowed apically. Therefore, we suggest *S.dentatus* should also be included in the *S.harmandi* species-group.

### Stenothemus acuticollis

Y. Yang & X. Yang sp. n.

DBEF4A52-1284-55F3-84CB-F2C4AF93C444

B8568E26-718F-4CE6-87F8-512F33D3B1FA

#### Materials

- **Type status:**Holotype. **Occurrence:** recordedBy: Lin Meiying; individualCount: 1; sex: male; lifeStage: adult; **Taxon:** scientificName: *Stenothemusacuticollis*; **Location:** country: China; stateProvince: Yunnan; locality: Tengchong, Wuhexiang, Zhengding; verbatimElevation: 1873 m; verbatimCoordinateSystem: 24°51′ N, 98°44′ E; **Record Level:** collectionCode: Insects; ownerInstitutionCode: IZAS; basisOfRecord: Preserved Specimen
- **Type status:**Paratype. **Occurrence:** recordedBy: Lin Meiying; individualCount: 8; sex: females; lifeStage: adult; **Taxon:** scientificName: *Stenothemusacuticollis*; **Location:** country: China; stateProvince: Yunnan; locality: Tengchong, Wuhexiang, Zhengding; verbatimElevation: 1873 m; verbatimCoordinates: 24°51′ N, 98°44′ E; **Record Level:** collectionCode: Insects; ownerInstitutionCode: IZAS; basisOfRecord: Preserved Specimen
- **Type status:**Paratype. **Occurrence:** recordedBy: Dong Yanju; individualCount: 1; sex: male; lifeStage: adult; **Taxon:** scientificName: *Stenothemusacuticollis*; **Location:** country: China; stateProvince: Yunnan; locality: Mangkuan, Baihualing; **Event:** eventDate: 02.03.2013; **Record Level:** collectionCode: Insects; ownerInstitutionCode: MHBU; basisOfRecord: Preserved Specimen
- **Type status:**Paratype. **Occurrence:** recordedBy: Sun Kai & Li Zhipeng; individualCount: 1; sex: male; lifeStage: adult; **Taxon:** scientificName: *Stenothemusacuticollis*; **Location:** country: China; stateProvince: Yunnan; locality: Gongshan, Dulongjiang; **Event:** eventDate: 14.08.2018; **Record Level:** collectionCode: Insects; ownerInstitutionCode: MHBU; basisOfRecord: Preserved Specimen
- **Type status:**Paratype. **Occurrence:** recordedBy: Xu Jishan & Zhang Jianxiong; individualCount: 1; sex: male; lifeStage: adult; **Taxon:** scientificName: *Stenothemusacuticollis*; **Location:** country: China; stateProvince: Yunnan; locality: Zhenyuan, Jiujia; **Record Level:** collectionCode: Insects; ownerInstitutionCode: MHBU; basisOfRecord: Preserved Specimen

#### Description

Body length (both sexes): 6.4‒8.1 mm (6.5 mm in holotype); width: 1.5‒2.3 mm (1.5 mm in holotype).

**Male** (Fig. 1c).

Body pale yellow mixed with irregular dark brown markings, except head black, with a reddish-brown marking on midline of vertex, apex of each antennomere yellow. Body densely covered with pale yellow pubescence, mixed with semi-recumbent pale yellow pubescence.

Head. Surface densely punctate, each side with a smooth, rectangular impression behind antennal fossa; eyes strongly protruding, head across eyes nearly as wide as pronotum; terminal maxillary palpomeres elongate-triangular, widest at mid-length; antennae filiform, extending to three-quarter length of elytra, antennomeres II 2.8 times as long as wide at apices, III 1.1 times longer than II, V longest, VI to X gradually shortened, XI longer than X, pointed at apex.

Pronotum. 1.1 times wider than long, anterior margin arcuate, anterior angles rounded, lateral margins arcuate, posterior margin bisinuate and narrowly bordered, posterior angles sharp, protruding, disc strongly convex on postero-lateral parts, surface densely punctate.

Elytra. 3.0 times as long as combined humeral width, 4.2 times longer than pronotum, lateral margins diverging posteriorly, surface densely punctuate, longitudinal costae hardly visible.

**Figure 2.**
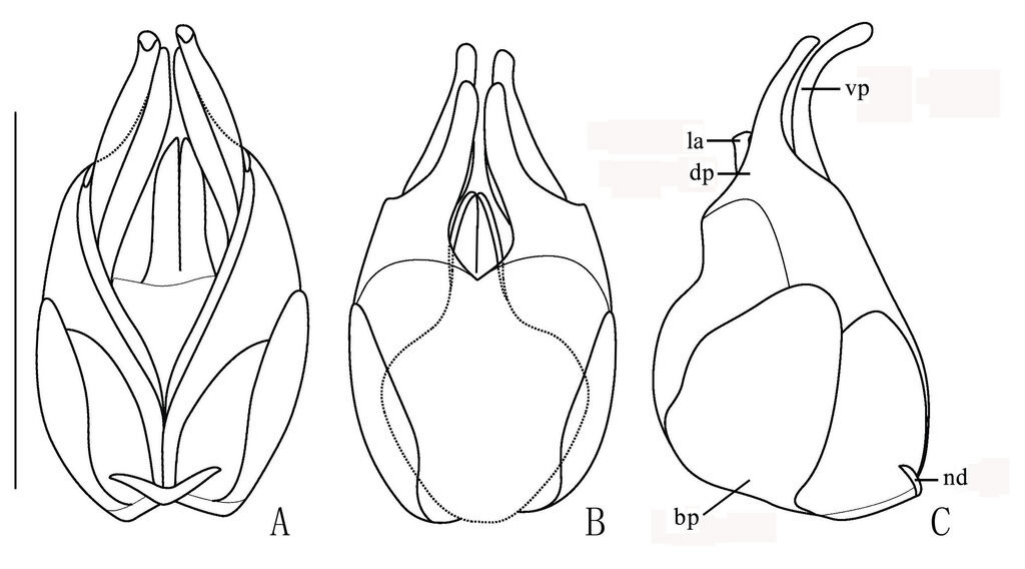
Aedeagus of *S.acuticollis* sp. n.: **A.** ventral view; **B.** dorsal view; **C.** lateral view; bp: basal piece; dp: dorsal plates; la: laterophyse; nd: nodule; vp: ventral processes of parameres; ag: accessory gland; Scale bar: 1.0 mm

Aedeagus (Fig. 2). Strongly swollen dorsally at base in lateral view, strongly reduced in diameter apically; basal piece (bp, phallobase) nearly as long as dorsal plate (dp) of each paramere, with a large, bifurcate conjoined middle nodule (nd) at base of ventral side; ventral processes (vp) of parameres approaching each other in ventral view, long and thickened apically, bent ventrally at an angle of about 60 degrees to median lobe in lateral view; dorsal plates shorter than ventral processes, greatly narrowed apically (apical part about one-third as wide as basal part); laterophyses (la) rounded at apices, exceeding into emargination between dorsal plates.

**Female**.

Body stouter than in male, eyes smaller, head across eyes about 0.9 times width of pronotum, antennae shorter and approximately extending to elytral mid-length; elytra with lateral margins diverging posteriorly more strongly in dorsal view.

Internal organ of reproductive system (Fig. 4a). Vagina (va) elongate, with median oviduct (ov) situated at ventro-apical part, vagina abruptly narrowed in apical part and extended into a short tube from where diverticulum and spermathecal duct are arising; diverticulum (di) short, 0.1 times as long as adult body length, evenly thinned apically, slender tube-shaped and spiral; spermathecal duct (sd) 0.1 times as long as diverticulum; spermatheca (sp) slender tube-shaped and spiral, thinner than spermathecal duct and 1.1 times as long as diverticulum, with basal part extended into a short tube, at opening of accessory gland (ag). Accessory gland thin in basal part and the remainder relatively thick, 1.6 times longer than spermatheca.

Abdominal sternite VIII (Fig. 6a, b). Obliquely narrowed posteriorly, latero-apical angles rounded, posterior margin shallowly and roundly emarginate in the middle and arcuate on both sides, present behind the notch with a membrane which is sclerotised.

#### Diagnosis

Most similar to *S.harmandi* (Bourgeois, 1902) in the shapes of pronotum and ventral process of each paramere of the aedeagus, but differs in the aedeagus which is strongly swollen dorsally at base in lateral view, with the dorsal plate of each paramere being abruptly narrowed apically in dorsal view. Unlike in the latter, the aedeagus is moderately swollen at the basal part and the dorsal plate of each paramere is evenly narrowed apically (Yang et al. 2014: figs. 21‒23). For the female, abdominal sternite VIII is shallowly emarginate in the middle of posterior margin, while deeply emarginate in *S.harmandi* (Yang et al. 2014: fig.11).

It also resembles *S.fugongensis* Y. Yang et X. Yang, 2014 in the body size and colouration and the shape of ventral process of each paramere of the aedeagus, but can be easily distinguished from *S.fugongensis* by the pronotum with sharp posterior angles and the aedeagus has the dorsal plate of each paramere abruptly narrowed apically in dorsal view with the laterophyses being rounded at the apices in lateral view. In comparison, *S.fugongensis* has the pronotum with rounded posterior angles (Yang et al. 2014: fig. 3) and the aedeagus has the dorsal plate of each paramere evenly narrowed apically, with the laterophyses hooked at the apices in lateral view (Yang et al. 2014: figs. 24‒26).

#### Etymology

The specific name is derived from the Latin *acutus* (sharp) and *collum* (neck), referring to its pronutum with sharp posterior angles.

#### Distribution

China (Yunnan).

### Stenothemus nigricolor

Y. Yang & S. Ge sp. n.

EBBF7B01-088F-5A95-8E0E-5BE5FEE7BE02

0DA06148-F090-4366-B3D5-06DB5FDBEF18

#### Materials

- **Type status:**Holotype. **Occurrence:** recordedBy: Qiu Tengfei; individualCount: 1; sex: male; lifeStage: adult; **Taxon:** scientificName: *Stenotemusnigricolor*; **Location:** country: China; stateProvince: Xizang; locality: Nyingchi, Mêdog, Dagmo, 81 K; **Event:** eventDate: 10.08.2016; **Record Level:** collectionCode: Insects; ownerInstitutionCode: MHBU; basisOfRecord: Preserved Specimen
- **Type status:**Paratype. **Occurrence:** recordedBy: Qiu Tengfei; individualCount: 3; sex: males; lifeStage: adult; **Taxon:** scientificName: *Stenotemusnigricolor*; **Location:** country: China; stateProvince: Xizang; locality: Nyingchi, Mêdog, Dagmo, 81 K; **Event:** eventDate: 10.08.2016; **Record Level:** collectionCode: Insects; ownerInstitutionCode: MHBU; basisOfRecord: Preserved Specimen
- **Type status:**Paratype. **Occurrence:** recordedBy: Qiu Tengfei; individualCount: 6; sex: females; lifeStage: adult; **Taxon:** scientificName: *Stenotemusnigricolor*; **Location:** country: China; stateProvince: Xizang; locality: Nyingchi, Mêdog, Dagmo, 81 K; **Event:** eventDate: 10.08.2016; **Record Level:** collectionCode: Insects; ownerInstitutionCode: MHBU; basisOfRecord: Preserved Specimen

#### Description

Body length (both sexes): 5.1‒6.5 mm (5.6 mm in holotype); width: 1.3‒1.8 mm (1.3 mm in holotype).

**Male** (Fig. 1d).

Body black, mouth-parts pale yellow, legs dark brown. Body densely covered with short, semi-recumbent pale pubescence.

Head. Surface densely punctate, each side with a smooth, rectangular impression behind antennal fossa; eyes small, strongly protruding, head across eyes 1.1 times wider than pronotum; terminal maxillary palpomeres elongate-triangular, widest at basal one-third; antennae filiform, extending to two-thirds length of elytra, antennomeres II 2.2 times as long as wide at apices, III 1.5 times longer than II, IV nearly as long as V, XI longer than X, pointed at apex.

Pronotum. 1.1 times wider than long, anterior margin arcuate, anterior angles rounded, lateral margins arcuate, posterior margin bisinuate and narrowly bordered, posterior angles sharp, protruding laterad, disc strongly convex on posterolateral parts, surface sparsely punctate.

Elytra. Nearly parallel-sided, 3.3 times as long as combined humeral width, 4.2 times as long as pronotum, surface densely punctate, longitudinal costae hardly visible.

**Figure 3.**
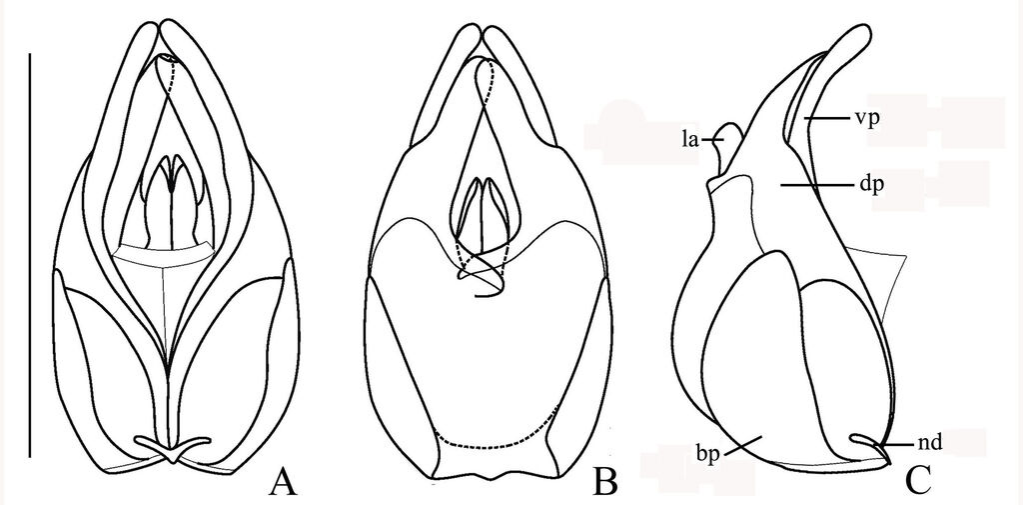
Aedeagus of *S.nigricolor* sp. n.: **A.** ventral view; **B.** dorsal view; **C.** lateral view; bp: basal piece; dp: dorsal plates; la: laterophyse; nd: nodule; vp: ventral processes of parameres; ag: accessory gland; Scale bar: 1.0 mm

Aedeagus (Fig. 3). Moderately swollen dorsally at base in lateral view, strongly reduced in diameter apically; basal piece nearly as long as dorsal plate of each paramere, with a large, bifurcate conjoined middle nodule at base of ventral side; ventral processes of parameres approaching each other in ventral view, long and thickened apically, bent ventrally at an angle of 30 degrees to median lobe in lateral view; dorsal plates shorter than ventral processes, abruptly narrowed apically (apical part about one-half as wide as basal part); laterophyses rounded at apices, exceeding into emargination between dorsal plates.

**Female**.

Body stouter than in male, eyes smaller, head across eyes nearly as long as pronotum, antennae shorter and approximately extending to quarter length of elytra; elytra 3.1 times as long as combined humeral width.

Internal organ of reproductive system (Fig. 4b). Vagina stout, with median oviduct situated at ventro-apical part, vagina abruptly narrowed in apical part and extended into a short tube from where diverticulum and spermathecal duct are arising; diverticulum long, 0.3 times as long as adult body length, evenly thinned apically, slender tube-shaped and spiral; spermathecal duct 0.1 times as long as diverticulum; spermatheca slender tube-shaped and spiral, thinner than spermathecal duct and 0.9 times longer than diverticulum, with basal part extended into a short tube, at opening of accessory gland. Accessory gland thin in basal part and the remainder relatively thick, 0.5 times as long as spermatheca.

Abdominal sternite VIII (Fig. 6c, d). Lateral margins narrowed posteriorly, latero-apical angles obtusely rounded, posterior margin shallowly emarginate, with a protuberance in the middle and arcuate on both sides, behind the notch with a membrane which is sclerotised.

#### Diagnosis

It resembles *S.alexandrae* Švihla, 2004 **stat. n.** (type locality: N. India) in the structure of aedeagus, but differs in the following characters: the body is black, pronotum is 1.1 times as wide as long, of which anterior margin is arcuate, the aedeagus has the ventral process of each paramere even in width in ventral view. In comparison, *S.alexandrae* has a dark brown body, pronotum is 1.4 times as wide as long, of which anterior margin is nearly straight and the ventral process of each paramere is widened apically (Švihla 2004: figs. 142‒144).

#### Etymology

The specific name is derived from the Latin *niger* (black) and *color* (colour), referring to its black body colouration.

#### Distribution

China (Xizang).

### Stenothemus distortirudis

Y. Yang & X. Yang, 2014

B97726FA-6B21-5EDA-96F8-AD00059E4426

Stenothemus distortirudis Y. Yang & X. Yang, 2014: 212, figs. 4, 16, 27‒29 and 44.

#### Materials

- **Type status:**Other material. **Occurrence:** recordedBy: Qiu Tengfei; individualCount: 1; sex: male; lifeStage: adult; **Taxon:** scientificName: *Stenothemusdistortirudis*; **Location:** country: China; stateProvince: Xizang; locality: Nyingchi, Mêdog, Baibung, Gelin; **Event:** eventDate: 04.08.2016; **Record Level:** collectionCode: Insects; ownerInstitutionCode: MHBU; basisOfRecord: Preserved Specimen
- **Type status:**Other material. **Occurrence:** recordedBy: Qiu Tengfei; individualCount: 1; sex: female; lifeStage: adult; **Taxon:** scientificName: *Stenothemusdistortirudis*; **Location:** country: China; stateProvince: Xizang; locality: Nyingchi, Mêdog, Baibung, Gelin; **Event:** eventDate: 04.08.2016; **Record Level:** collectionCode: Insects; ownerInstitutionCode: MHBU; basisOfRecord: Preserved Specimen

#### Description

**Female.** Internal organ of reproductive system (Fig. 4c). Vagina elongate, with median oviduct situated at ventro-apical part, vagina abruptly narrowed in apical part and extended into a short tube from where diverticulum and spermathecal duct are arising; diverticulum long, 0.4 times as long as adult body length, evenly thinned apically, slender tube-shaped and spiral; spermathecal duct 0.2 times as long as diverticulum; spermatheca slender tube-shaped and spiral, thinner than spermathecal duct and nearly as long as diverticulum, with basal part extended into a short tube, at opening of accessory gland. Accessory gland thin in basal part and the remainder relatively thick, nearly as long as spermatheca.

#### Distribution

China (Xizang).

### Stenothemus laticollis

Y. Yang & X. Yang, 2014

5EF4A01D-DF62-5EBC-B681-12394494FDDD

Stenothemus laticollis Y. Yang & X. Yang, 2014: 217, figs. 8, 20, 39‒41 and 43.

#### Materials

- **Type status:**Other material. **Occurrence:** recordedBy: Shi Fuming; individualCount: 1; sex: male; lifeStage: adult; **Taxon:** scientificName: *Stenothemuslaticollis*; **Location:** country: China; stateProvince: Xizang; locality: Nyingchi, Dongjug; **Event:** eventDate: 28.09.2007; **Record Level:** collectionCode: Insects; ownerInstitutionCode: MHBU; basisOfRecord: Preserved Specimen
- **Type status:**Other material. **Occurrence:** recordedBy: Shi Fuming; individualCount: 1; sex: female; lifeStage: adult; **Taxon:** scientificName: *Stenothemuslaticollis*; **Location:** country: China; stateProvince: Xizang; locality: Nyingchi, Dongjug; **Event:** eventDate: 28.09.2007; **Record Level:** collectionCode: Insects; ownerInstitutionCode: MHBU; basisOfRecord: Preserved Specimen
- **Type status:**Other material. **Occurrence:** recordedBy: Song Zhishun; individualCount: 1; sex: male; lifeStage: adult; **Taxon:** scientificName: *Stenothemuslaticollis*; **Location:** country: China; stateProvince: Xizang; locality: Nyingchi, Pêlong; verbatimElevation: 2100 m; **Event:** eventDate: 01.09.2005; **Record Level:** collectionCode: Insects; ownerInstitutionCode: IZAS; basisOfRecord: Preserved Specimen
- **Type status:**Other material. **Occurrence:** recordedBy: Chen Xiaolin; individualCount: 1; sex: female; lifeStage: adult; **Taxon:** scientificName: *Stenothemuslaticollis*; **Location:** country: China; stateProvince: Xizang; locality: Nyingchi, Pêlong; verbatimElevation: 2100 m; **Event:** eventDate: 02.09.2005; **Record Level:** collectionCode: Insects; ownerInstitutionCode: IZAS; basisOfRecord: Preserved Specimen
- **Type status:**Other material. **Occurrence:** recordedBy: Han Yinheng; individualCount: 1; sex: male; lifeStage: adult; **Taxon:** scientificName: *Stenothemuslaticollis*; **Location:** country: China; stateProvince: Xizang; locality: Bomi, Yi’ong; verbatimElevation: 2300 m; **Event:** eventDate: 14.08.1983; **Record Level:** collectionCode: Insects; ownerInstitutionCode: IZAS; basisOfRecord: Preserved Specimen
- **Type status:**Other material. **Occurrence:** recordedBy: Wang Xuejian; individualCount: 1; sex: male; lifeStage: adult; **Taxon:** scientificName: *Stenothemuslaticollis*; **Location:** country: China; stateProvince: Xizang; locality: Nyingchi, Mainling, Nanyigou; verbatimElevation: 3173 m; **Event:** eventDate: 04.09.2005; **Record Level:** collectionCode: Insects; ownerInstitutionCode: IZAS; basisOfRecord: Preserved Specimen

#### Description

**Female.** Internal organ of reproductive system (Fig. 4d). Vagina elongate, with median oviduct situated at ventro-apical part, vagina abruptly narrowed in apical part and extended into a long tube from where diverticulum and spermathecal duct are arising; diverticulum long, 0.3 times as long as adult body length, evenly thinned apically, slender tube-shaped and spiral; spermathecal duct 0.2 times as long as diverticulum; spermatheca slender tube-shaped and spiral, thinner than spermathecal duct and 1.1 times longer than diverticulum, with basal part extended into a short tube, at opening of accessory gland. Accessory gland thin in basal part and the remainder relatively thick, 0.8 times as long as spermatheca.

#### Distribution

China (Xizang).

### Stenothemus leishanensis

Y. Yang & X. Yang, 2014

4E30D21E-DD74-565B-8BA2-5DFC730C6192

Stenothemus leishanensis Y. Yang & X. Yang, 2014: 216, figs. 7, 19, 36‒38 and 44.

#### Materials

- **Type status:**Paratype. **Occurrence:** recordedBy: Wang Jiliang & Gao Chao; individualCount: 1; sex: female; lifeStage: adult; **Taxon:** scientificName: *Stenothemusleishanensis*; **Location:** country: China; stateProvince: Guizhou; locality: Leigongshan Forestry Centre; **Event:** eventDate: 13‒14.09.2005; **Record Level:** collectionCode: Insects; ownerInstitutionCode: MHBU; basisOfRecord: Preserved Specimen
- **Type status:**Other material. **Occurrence:** recordedBy: Wang Jiliang & Gao Chao; individualCount: 3; sex: males; lifeStage: adult; **Taxon:** scientificName: *Stenothemusleishanensis*; **Location:** country: China; stateProvince: Guizhou; locality: Leigongshan Forestry Centre; **Event:** eventDate: 13‒14.09.2005; **Record Level:** collectionCode: Insects; ownerInstitutionCode: MHBU; basisOfRecord: Preserved Specimen

#### Description

**Female.** Internal organ of reproductive system (Fig. 5a). Vagina elongate, with median oviduct situated at ventro-apical part, vagina abruptly narrowed in apical part and extended into a short tube from where diverticulum and spermathecal duct are arising; diverticulum short, 0.2 times as long as adult body length, evenly thinned apically, slender tube-shaped and spiral; spermathecal duct 0.1 times longer than diverticulum; spermatheca slender tube-shaped and spiral, thinner than spermathecal duct and nearly as long as diverticulum, with basal part extended into a short tube, at opening of accessory gland. Accessory gland thin in basal part and the remainder relatively thick, 0.9 times as long as spermatheca.

#### Distribution

China (Guizhou).

### Stenothemus orbiculatus

Švihla, 2005

C042A5BB-1CEB-5CF6-A81F-5C4BE998393E

Stenothemus orbiculatus Švihla, 2005: 99, figs. 58, 62 and 65.

#### Materials

- **Type status:**Other material. **Occurrence:** recordedBy: Liang Hongbin; individualCount: 2; sex: females; lifeStage: adult; **Taxon:** scientificName: *Stenothemusorbiculatus*; **Location:** country: China; county: Xizang; locality: Mêdog, Zhamo Highway, 62 K; verbatimElevation: 2787 m; **Event:** eventDate: 29.08.2015; **Record Level:** collectionCode: Insects; ownerInstitutionCode: IZAS; basisOfRecord: Preserved Specimen
- **Type status:**Other material. **Occurrence:** recordedBy: Yao Jian; individualCount: 1; sex: male; lifeStage: adult; **Taxon:** scientificName: *Stenothemusorbiculatus*; **Location:** country: China; county: Xizang; locality: Mêdog, Zhamo Highway, 62 K; verbatimElevation: 2787 m; **Event:** eventDate: 29.08.2015; **Record Level:** collectionCode: Insects; ownerInstitutionCode: IZAS; basisOfRecord: Preserved Specimen
- **Type status:**Other material. **Occurrence:** recordedBy: Liang Hongbin & Wang Mingqiang; individualCount: 1; sex: male; lifeStage: adult; **Taxon:** scientificName: *Stenothemusorbiculatus*; **Location:** country: China; stateProvince: Xizang; locality: Mainling, Paimo Highway; verbatimElevation: 3321 m; **Event:** eventDate: 06.08.2015; **Record Level:** collectionCode: Insects; ownerInstitutionCode: IZAS; basisOfRecord: Preserved Specimen

#### Description

**Female.** Internal organ of reproductive system (Fig. 5b). Vagina elongate, with median oviduct situated at ventro-apical part, vagina abruptly narrowed in apical part and extended into a short tube from where diverticulum and spermathecal duct are arising; diverticulum short, 0.1 times as long as adult body length, evenly thinned apically, slender tube-shaped and spiral; spermathecal duct 0.2 times as long as diverticulum; spermatheca slender tube-shaped and spiral, thinner than spermathecal duct and 1.1 times longer than diverticulum, with basal part extended into a short tube, at opening of accessory gland. Accessory gland thin in basal part and the remainder relatively thick, nearly as long as spermatheca.

#### Distribution

China (Xizang), India.

### Stenothemus septimus

Y. Yang & X. Yang, 2014

82917064-57D1-52EF-AEF0-063E4BC6DF8B

Stenothemus septimus Y. Yang & X. Yang, 2014: 214, figs. 6, 18, 33‒35 and 43.

#### Materials

- **Type status:**Other material. **Occurrence:** recordedBy: Liang Hongbin; individualCount: 2; sex: males; lifeStage: adult; **Taxon:** scientificName: *Stenothemusseptimus*; **Location:** country: China; stateProvince: Xizang; locality: Mêdog, 80 K; verbatimElevation: 2129 m; **Event:** eventDate: 15.06.2016; **Record Level:** collectionCode: Insects; ownerInstitutionCode: IZAS; basisOfRecord: Preserved Specimen

#### Description

**Female.** Internal organ of reproductive system (Fig. 5c). Vagina elongate, with median oviduct situated at ventro-apical part, vagina abruptly narrowed in apical part and extended into a short tube from where diverticulum and spermathecal duct are arising; diverticulum short, 0.1 times as long as adult body length, evenly thinned apically, slender tube-shaped and spiral; spermathecal duct 0.3 times as long as diverticulum; spermatheca slender tube-shaped and spiral, thinner than spermathecal duct and 0.7 times longer than diverticulum, with basal part extended into a short tube, at opening of accessory gland. Accessory gland thin in basal part and the remainder relatively thick, 1.8 times as long as spermatheca.

#### Diagnosis

China (Xizang).

### Stenothemus subnitidus

Švihla, 2005

8C269164-6C16-5DCC-B6D9-419E0ADEB825

Stenothemus subnitidus Švihla, 2005: 97, figs 60, 63.

#### Materials

- **Type status:**Other material. **Occurrence:** recordedBy: Shi Aimin; individualCount: 1; sex: female; lifeStage: adult; **Taxon:** scientificName: *Stenothemussubnitidus*; **Location:** country: China; stateProvince: Xizang; locality: Zham; **Event:** eventDate: 27.07.2005; **Record Level:** collectionCode: Insects; ownerInstitutionCode: MHBU; basisOfRecord: Preserved Specimen

#### Description

**Female.** Internal organ of reproductive system (Fig. 5d). Vagina elongate, with median oviduct situated at ventro-apical part, vagina abruptly narrowed in apical part and extended into a short tube from where diverticulum and spermathecal duct are arising; diverticulum short, 0.2 times as long as adult body length, evenly thinned apically, slender tube-shaped and spiral; spermathecal duct 0.3 times as long as diverticulum; spermatheca slender tube-shaped and spiral, thinner than spermathecal duct and 1.4 times longer than diverticulum, with basal part extended into a short tube, at opening of accessory gland. Accessory gland thin in basal part and the remainder relatively thick, 1.1 times as long as spermatheca.

#### Distribution

China (Xizang), India?

## Identification Keys

### Key to the species of *Stenothemusharmandi* species-group

**Table:** 

1	Posterior angles of pronotum obtuse and rounded (Fig. 1b)	2
–	Posterior angles of pronotum sharp and protruding laterad (Fig. 1a, c, d)	4
2	Aedeagus: dorsal plate of each paramere evenly narrowed apically in dorsal view (Yang et al. 2014: fig. 25)	*S.fugongensis* Y. Yang & X. Yang, 2014
–	Aedeagus: dorsal plate of each paramere abruptly narrowed in the middle in dorsal view (Švihla 2004: fig. 145; Švihla 2005: fig. 62)	3
3	Aedeagus: ventral process of each paramere bent ventrad at an angle of about 45 degrees with median lobe in lateral view (Švihla 2005: fig. 65)	*S.orbiculatus* Švihla, 2005
–	Aedeagus: ventral process of each paramere bent ventrad at an angle of about 30 degrees with median lobe in lateral view (Wittmer 1974: fig. 5)	*S.dentatus* Wittmer, 1974
4	Body dark brown or black (Fig. 1a, d)	5
–	Body pale yellow, with dark brown markings on disc of pronotum, elytra and legs (Fig. 1b, c)	6
5	Pronotum about 1.1 times as wide as long, anterior margin arcuate, antennomeres IV‒XI cylindrically thickened (Fig. 1d), aedeagus: ventral process of each paramere even in width in ventral view (Fig. 3)	*S.nigricolor* sp.n.
–	Pronotum about 1.4 times as wide as long, anterior margin nearly straight, antennomeres IV‒XI flattened (Fig. 1a), aedeagus: ventral process of each paramere widened apically in ventral view (Švihla 2004: figs. 142‒144)	*S.alexandrae* Švihla, 2004 stat. n.
6	Aedeagus: ventral process of each paramere hardly thickened at apex, nearly uniform width on the whole in lateral view (Yang et al. 2014: figs. 33‒35)	*S.septimus* Y. Yang *&* X. Yang, 2014
–	Aedeagus: ventral process of each paramere thickened at apex, narrowed at base in lateral view (Švihla 2005: figs. 63‒64; Yang et al. 2014: figs. 23, 29, 32, 38, 41; Fig. 2)	7
7	Aedeagus: dorsal plate of each paramere evenly narrowed apically or nearly parallel-sided in dorsal view (Yang et al. 2014: figs. 22, 31)	8
–	Aedeagus: dorsal plate of each paramere abruptly narrowed apically in dorsal view (Švihla 2005: figs. 60‒61; Yang et al. 2014: figs. 28, 37, 40; Fig. 2)	9
8	Pronotum 1.1 times as long as wide (Yang et al. 2014: fig. 5); abdominal sternite VIII of female with posterior margin triangularly emarginate in the middle (Yang et al. 2014: fig. 17)	*S.parallelus* Y. Yang *&* X. Yang, 2014
–	Pronotum 1.2 times as long as wide (Yang et al. 2014: figs. 1‒2); abdominal sternite VIII of female with posterior margin roundly emarginate in the middle (Yang et al. 2021a: fig. 11)	*S.harmandi* (Bourgeois, 1902)
9	Aedeagus: ventral process of each paramere with the bent portion at apical part shorter than the basal portion in lateral view (Yang et al. 2014: figs. 29, 41)	10
–	Aedeagus: ventral process of each paramere with the bent portion at apical part longer than the basal portion in lateral view (Švihla 2005: figs. 63‒64; Yang et al. 2014: figs. 38; Fig. 2)	11
10	Aedeagus: ventral process of each paramere widened at the base, in a bent stick-shape in ventral view (Yang et al. 2014: figs. 27-29); female reproductive system: vagina extended into a short tube in apical part (Fig. 4c)	*S.distortirudis* Y. Yang & X. Yang, 2014
–	Aedeagus: ventral process of each paramere even in width at the base in ventral view (Yang et al. 2014: figs. 39-41); female reproductive system: vagina extended into a long tube in apical part (Fig. 4d)	*S.laticollis* Y. Yang & X. Yang, 2014
11	Aedeagus: ventral process of each paramere bent ventrad at an angle of less than 30 degrees with median lobe in lateral view (Yang et al. 2014: figs. 36‒38)	*S.leishanensis* Y. Yang & X. Yang, 2014
–	Aedeagus: ventral process of each paramere bent ventrad at an angle of over 45 degrees with median lobe in lateral view (Švihla 2005: figs. 63‒64; Fig. 2)	12
12	Aedeagus: ventral process of each paramere truncated at apex in lateral view (Švihla 2005: fig. 64)	*S.holosericus* Švihla, 2005
–	Aedeagus: ventral process of each paramere rounded at apex in lateral view (Švihla 2005: fig. 63; Fig. 2)	13
13	Aedeagus: dorsal plates converging to the middle part, then diverging towards apex in dorsal view (Švihla 2005: fig. 60); female reproductive system: spermathecal duct long (Fig. 5d)	*S.subnitidus* Švihla, 2005
–	Aedeagus: dorsal plates converging throughout from base to apex in dorsal view (Fig. 2); female reproductive system: spermathecal duct short (Fig. 4a)	*S.acuticollis* sp. n.

## Discussion

The present study first illustrates the female internal organ of the reproductive system for the *S.harmandi* speices-group. As in other *Stenothemus* species (Yang et al. 2021a, Yang et al. 2021b, Ge et al. 2021), the oviduct is suitated at the apical part of the vagina, the spermatheca is composed of only one spiral and thin tube and the diverticulum is thinly spiral-tubed. The combination of these characters could distinguish *Stenothemus* from other genera of Cantharinae, such as *Cantharis* L. (Li et al. 2016a), *Themus* Motschulsky (Yang et al. 2018, Yang et al. 2019a, Yang et al. 2019b), *Lycocerus* Gorham (Xi et al. 2021b, Xi et al. 2021a), *Cephalomalthinus* Pic and *Micropodabrus* Pic (Li et al. 2016b). However, we cannot summarise any common charater in this structure for this species-group to be distinguished from other well-known species (Yang et al. 2021a, Yang et al. 2021b, Ge et al. 2021). Maybe, with the discovery of new species in the near future, some more potential characters will be explored in defining the species-group.

## Supplementary Material

XML Treatment for Stenothemus harmandi

XML Treatment for Stenothemus alexandrae

XML Treatment for Stenothemus dentatus

XML Treatment for Stenothemus acuticollis

XML Treatment for Stenothemus nigricolor

XML Treatment for Stenothemus distortirudis

XML Treatment for Stenothemus laticollis

XML Treatment for Stenothemus leishanensis

XML Treatment for Stenothemus orbiculatus

XML Treatment for Stenothemus septimus

XML Treatment for Stenothemus subnitidus

## Figures and Tables

**Figure 1a.**
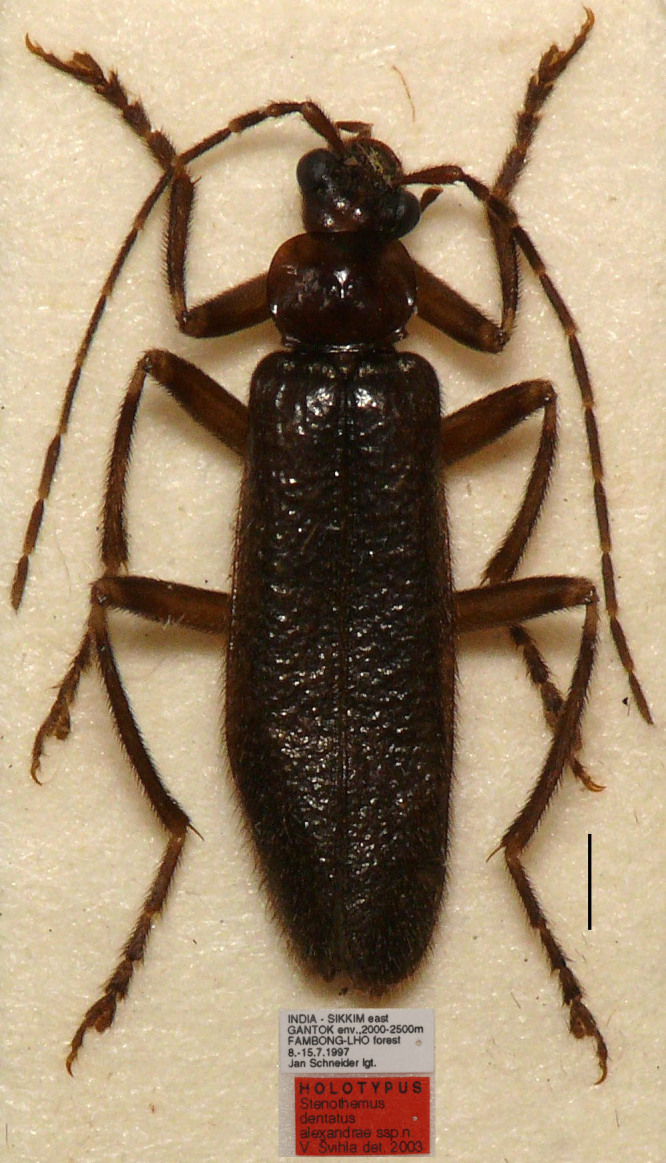
*S.alexandrae* Švihla, 2004 stat. n.

**Figure 1b.**
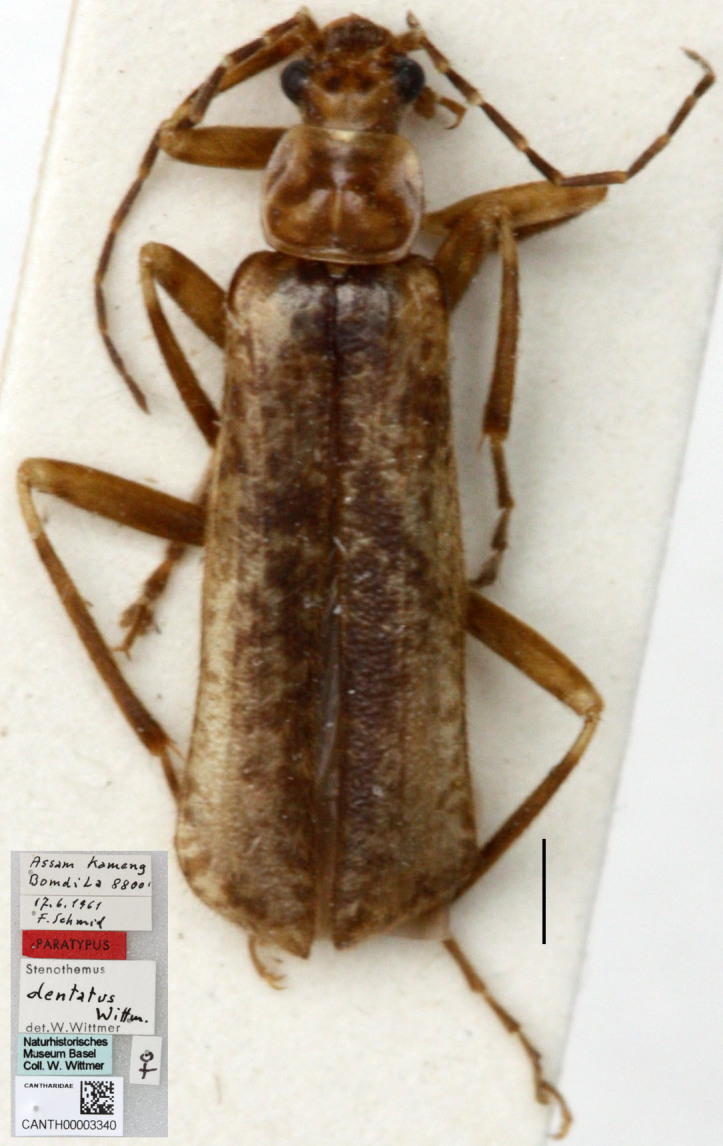
*S.dentatus* Wittmer, 1974

**Figure 1c.**
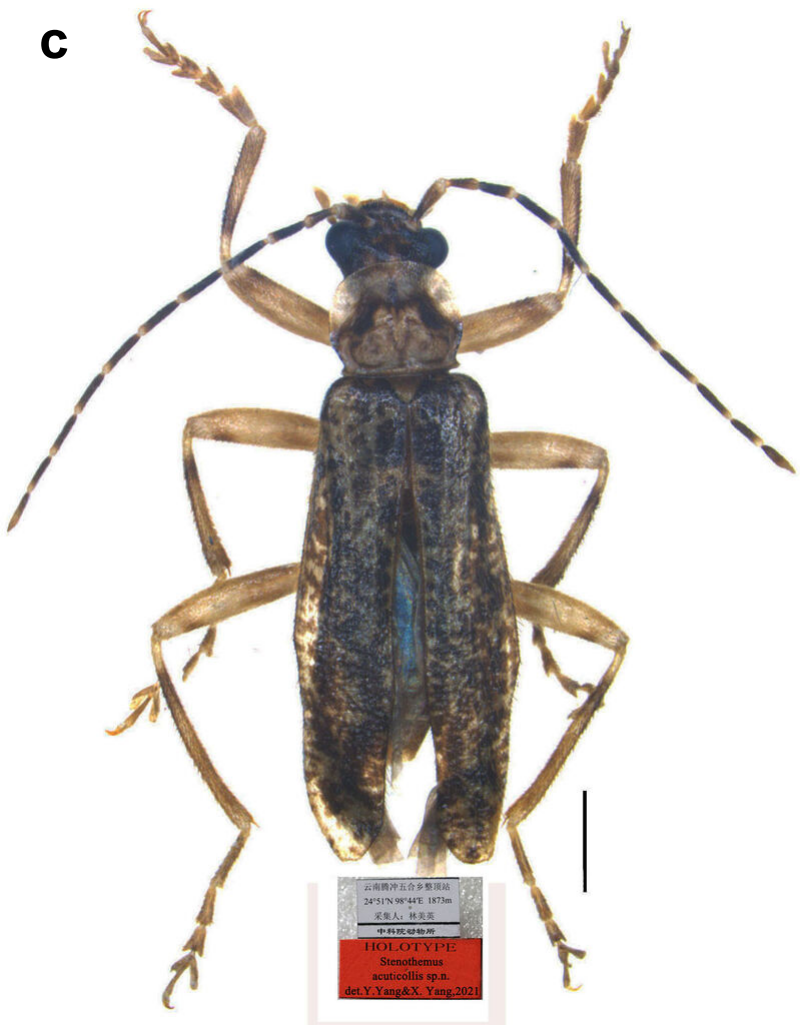
*S.acuticollis* sp. n.

**Figure 1d.**
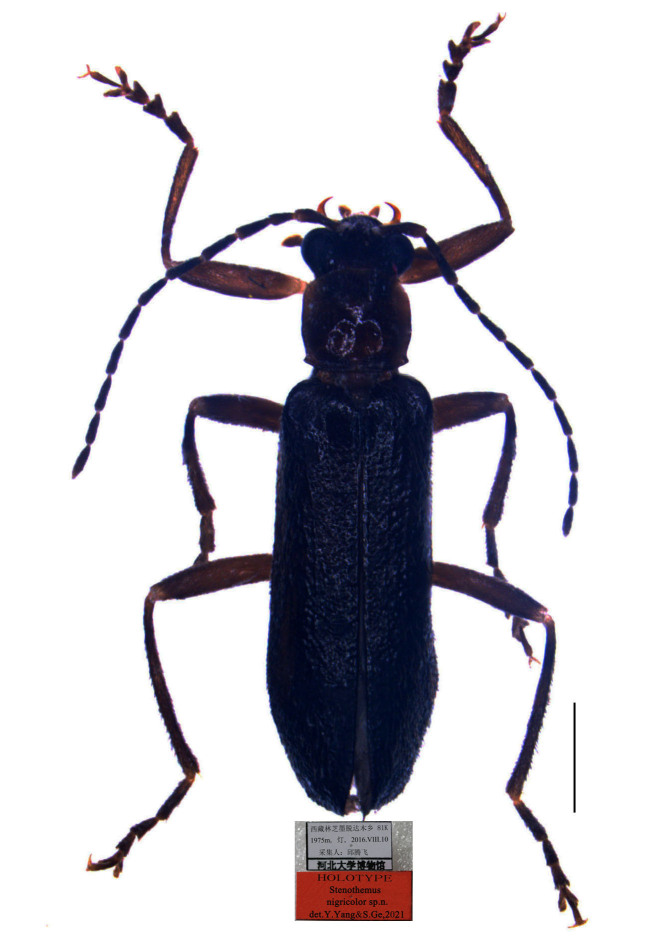
*S.nigricolor* sp. n.

**Figure 4a.**
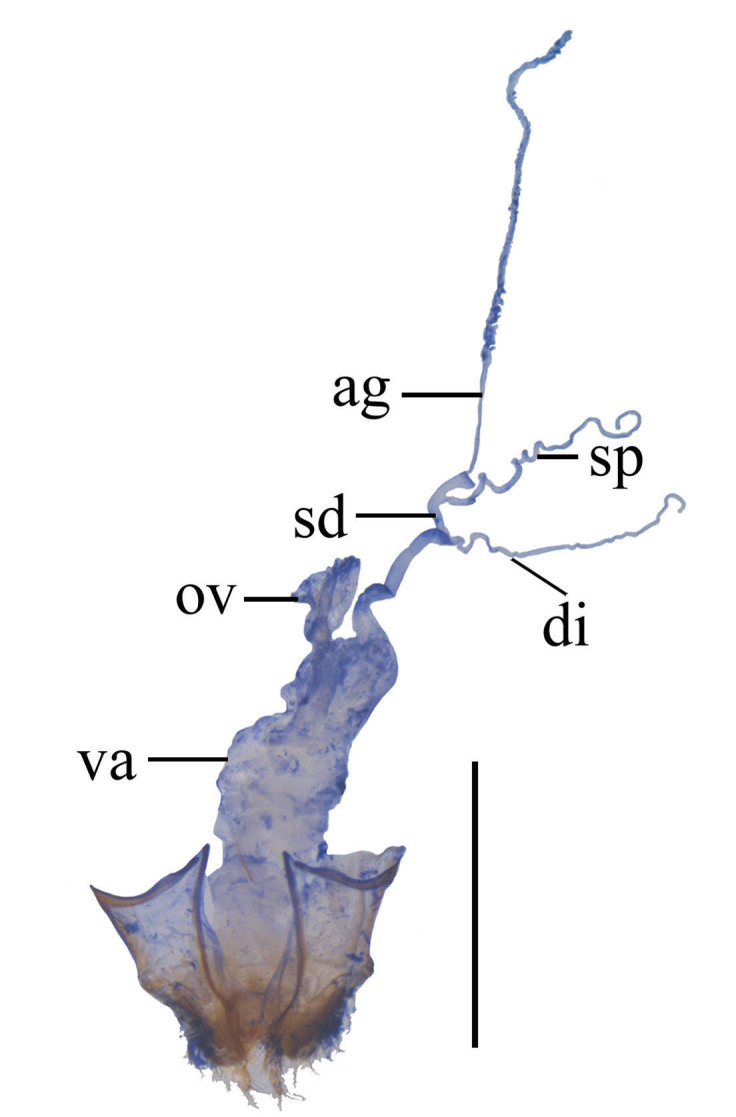
*S.acuticollis* sp. n.

**Figure 4b.**
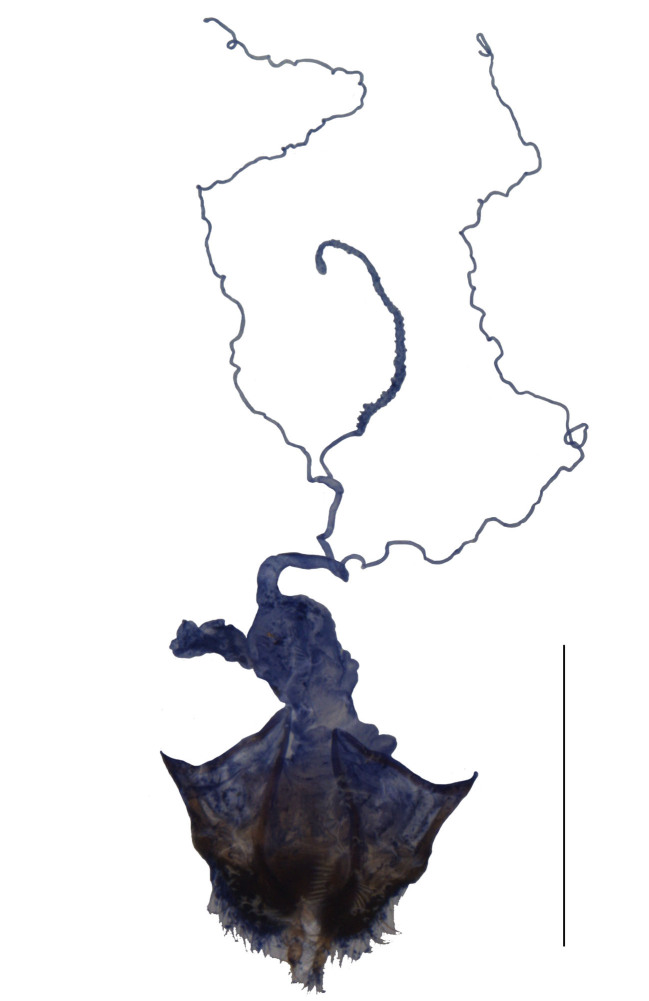
*S.nigricolor* sp. n.

**Figure 4c.**
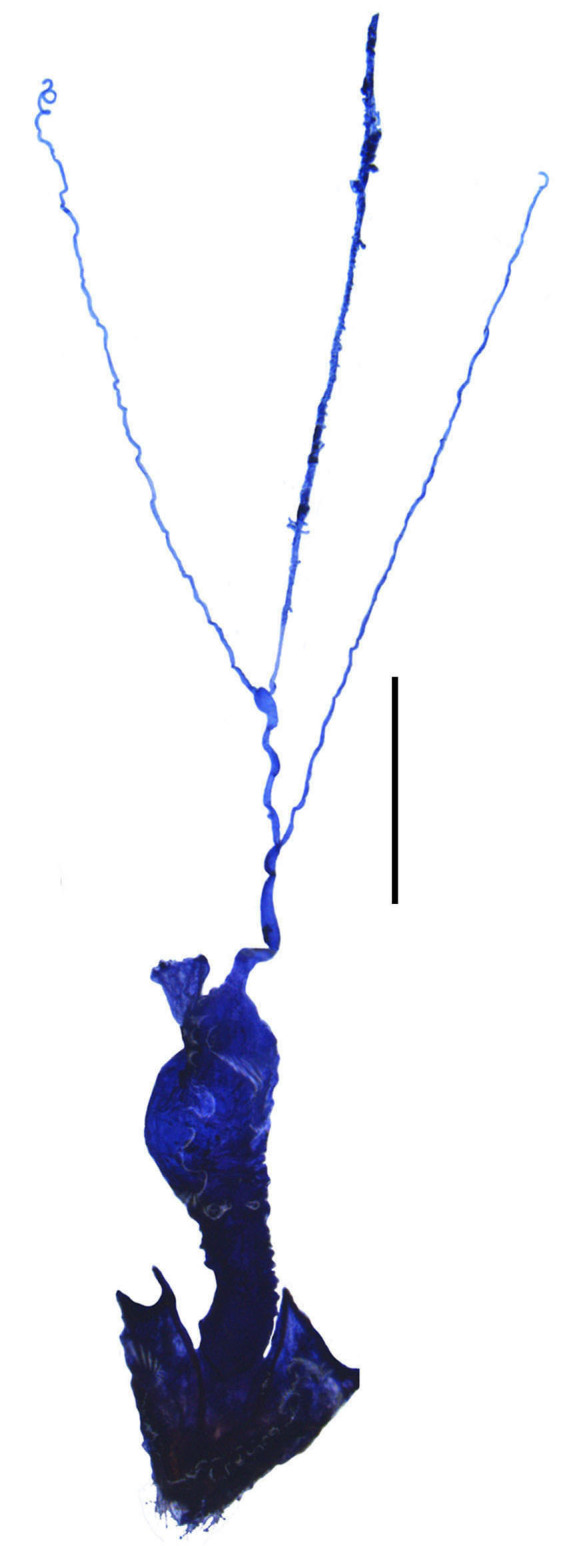
*S.distortirudis* Y. Yang & X. Yang, 2014

**Figure 4d.**
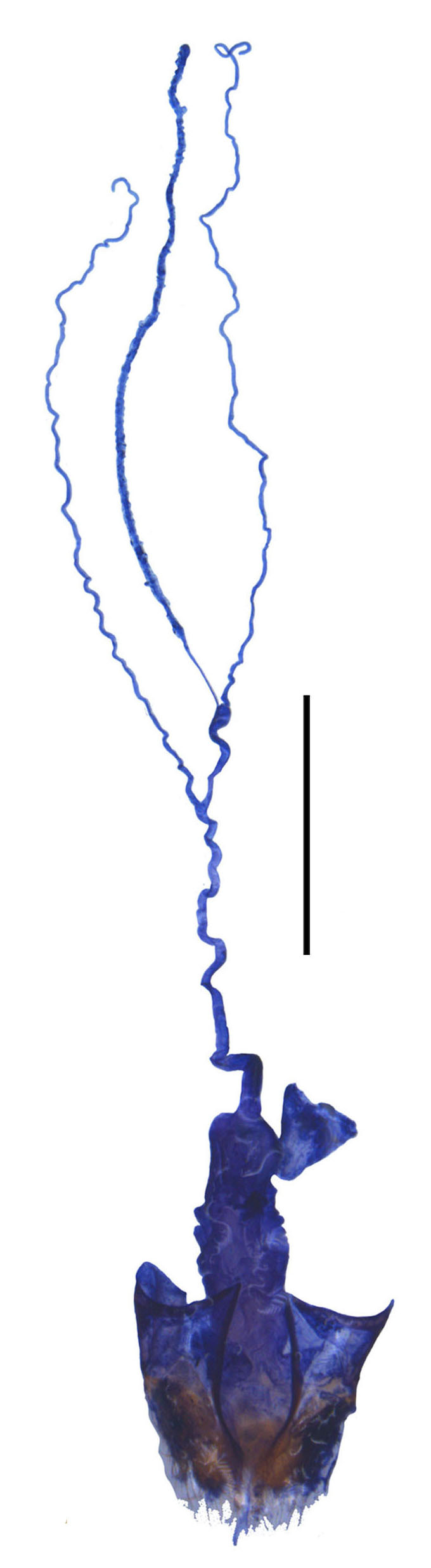
*S.laticollis* Y. Yang & X. Yang, 2014

**Figure 5a.**
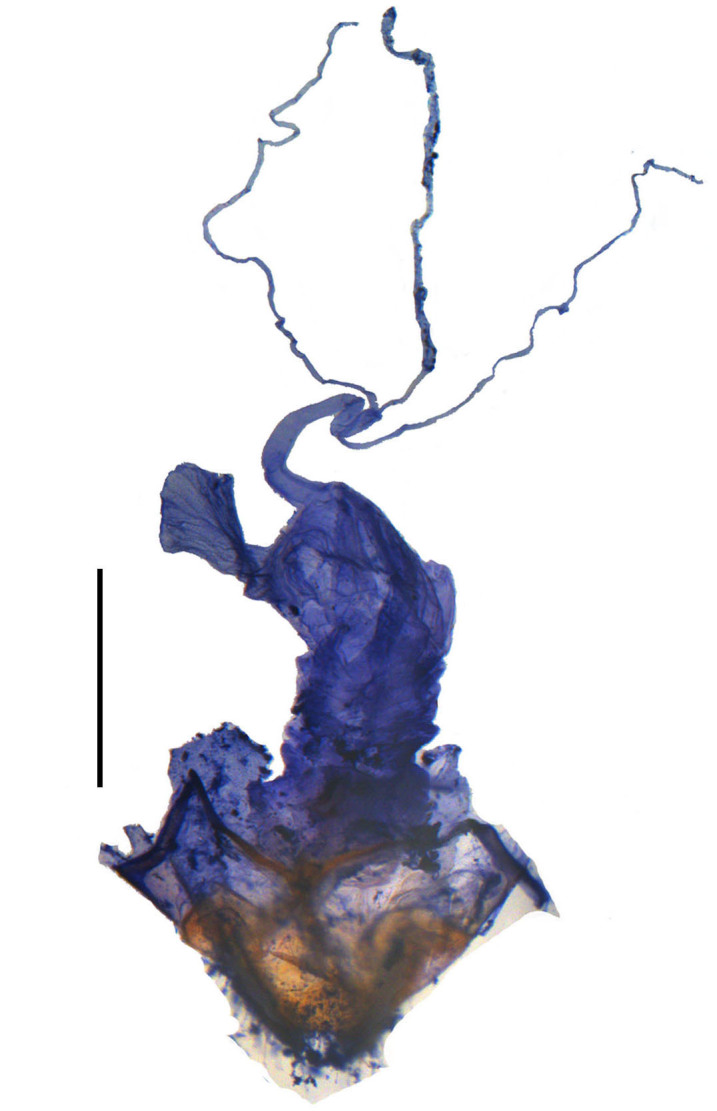
*S.leishanensis* Y. Yang & X. Yang, 2014

**Figure 5b.**
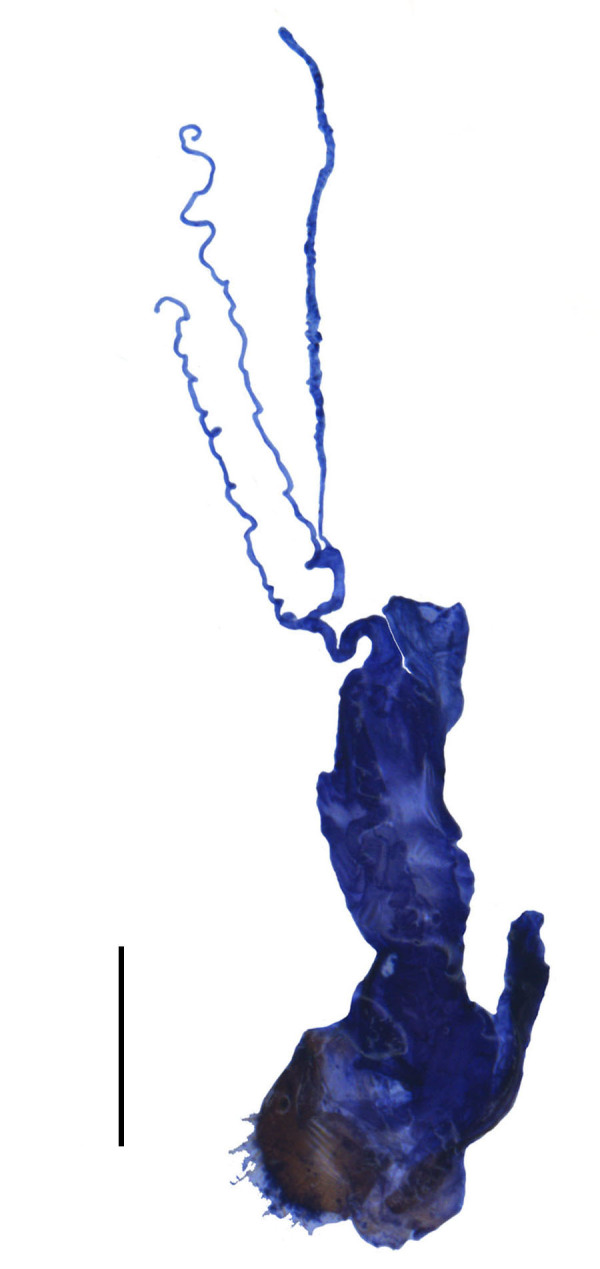
*S.orbiculatus* Švihla, 2005

**Figure 5c.**
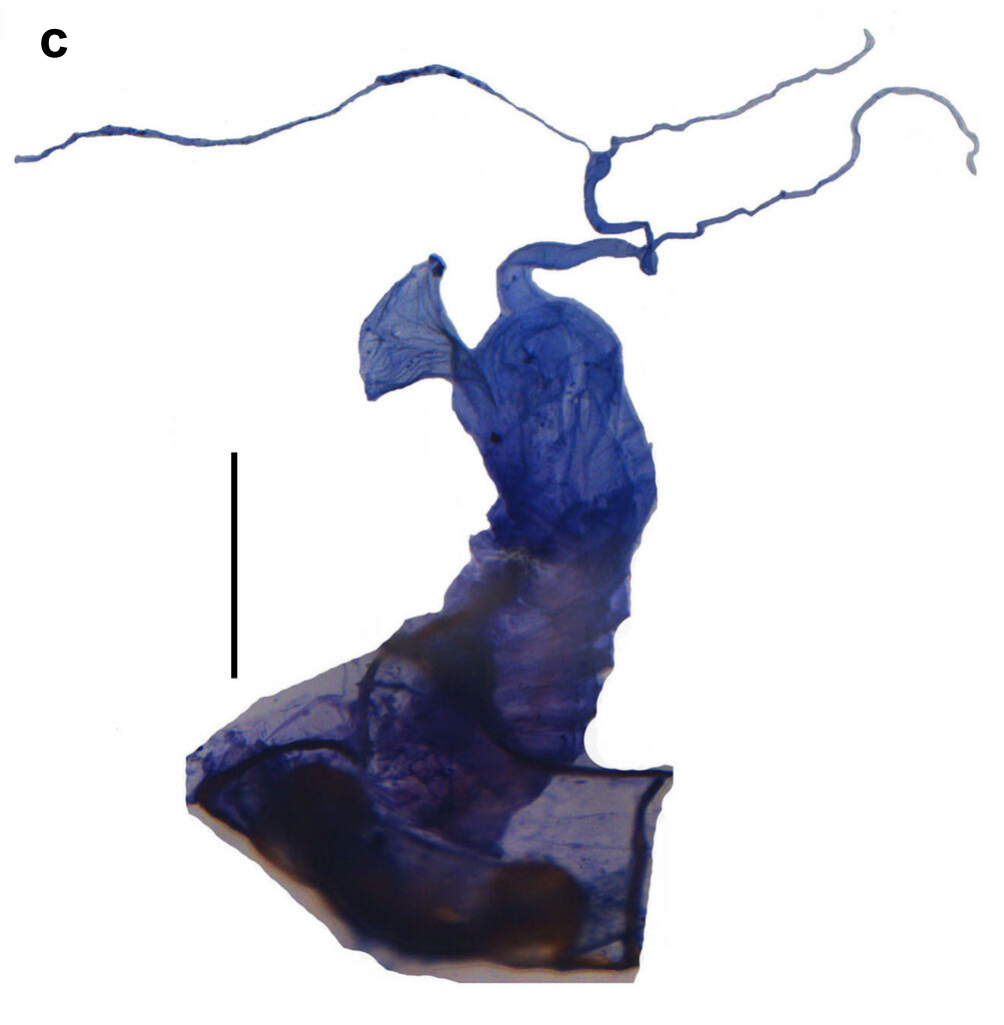
*S.septimus* Y. Yang & X. Yang, 2014

**Figure 5d.**
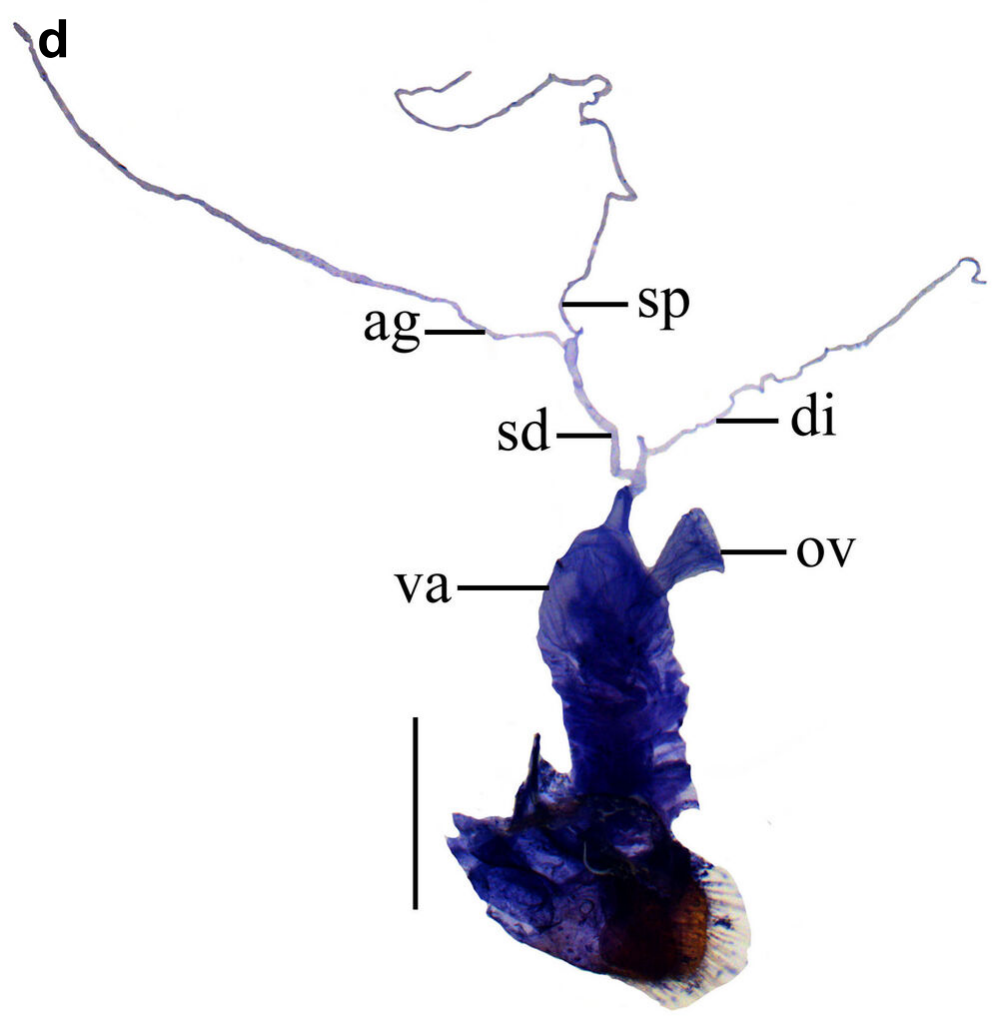
*S.subnitidus* Švihla, 2005

**Figure 6a.**
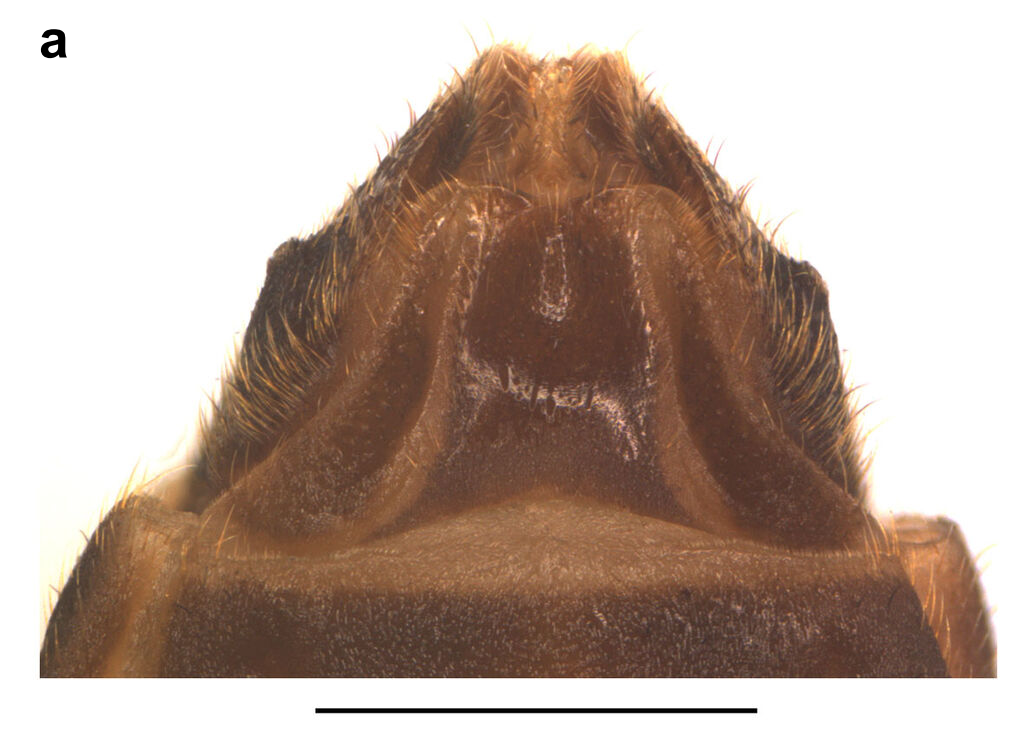
*S.acuticollis* sp.n., ventral view, in natural state

**Figure 6b.**
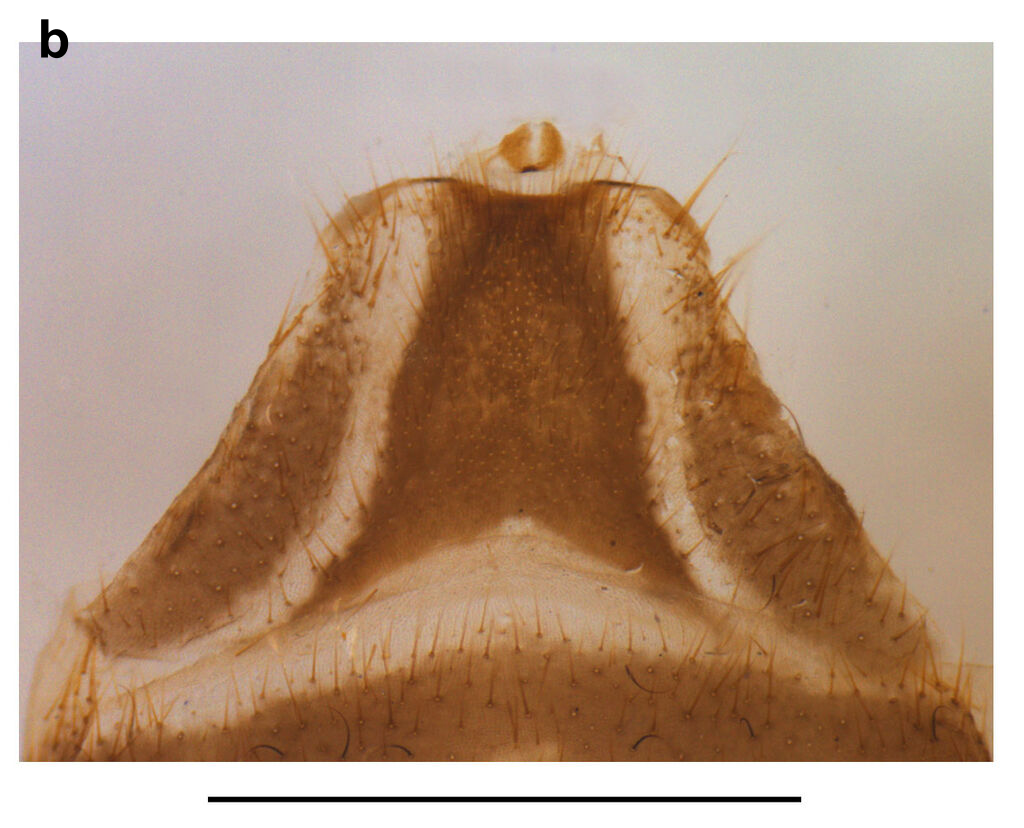
*S.acuticollis* sp.n., dorsal view, abdominal sternite VIII

**Figure 6c.**
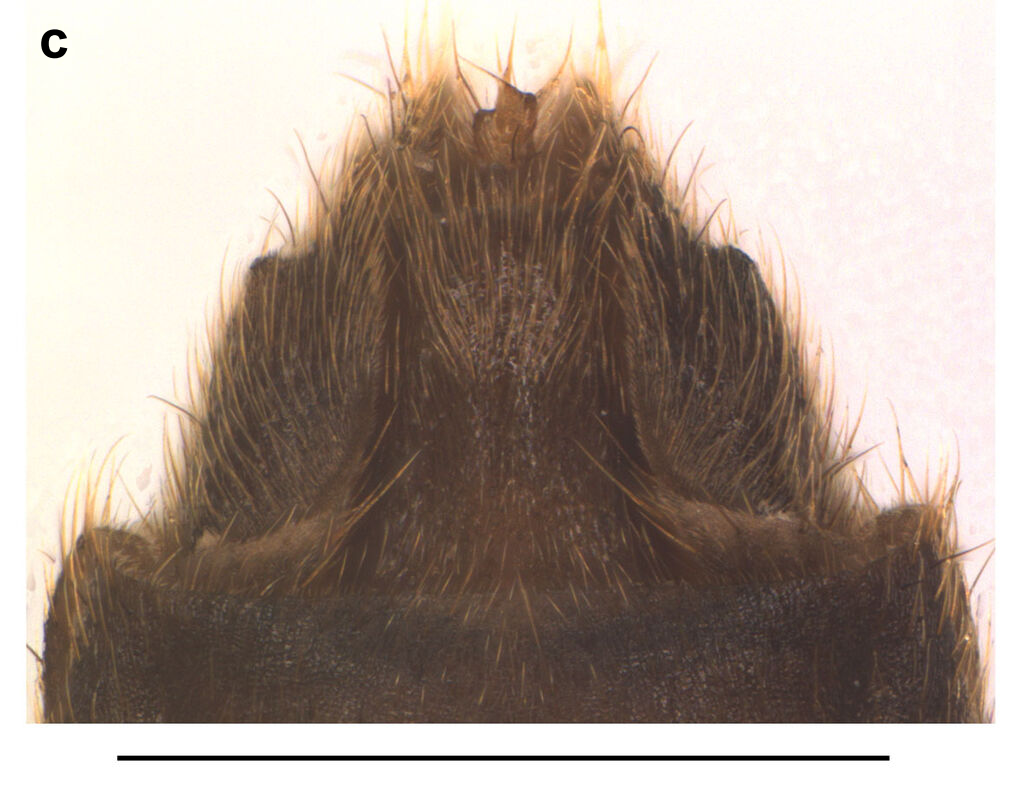
*S.nigricolor* sp.n., ventral view, in natural state

**Figure 6d.**
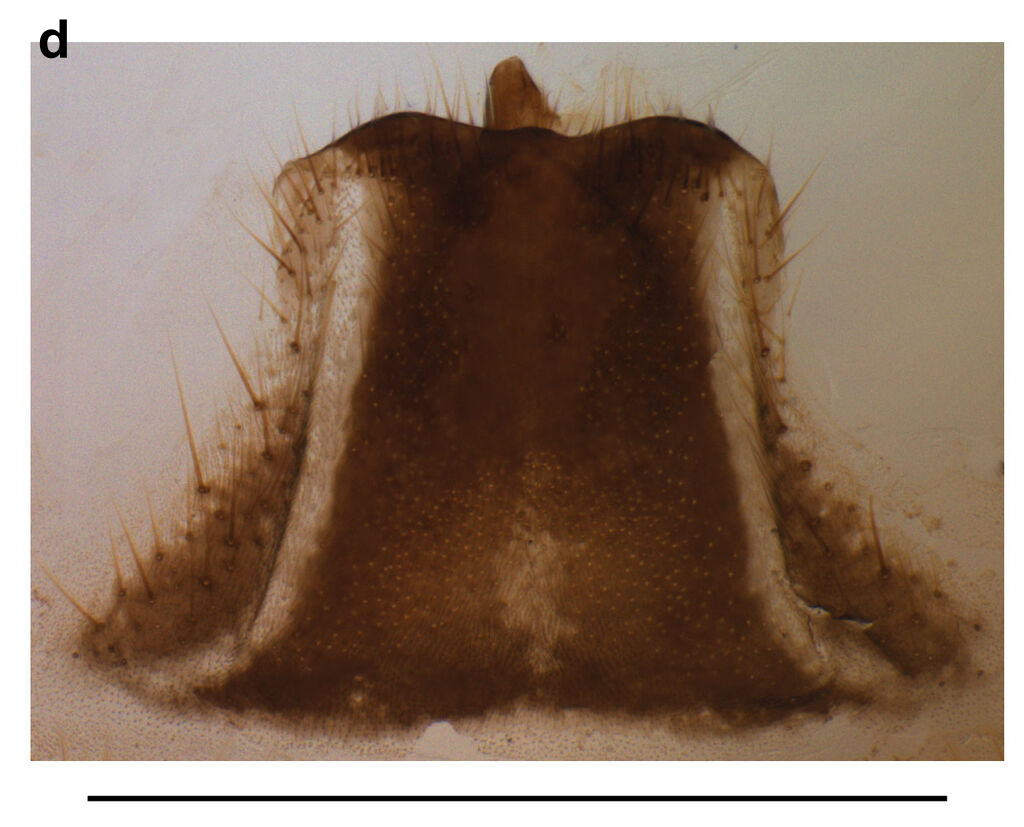
*S.nigricolor* sp.n., dorsal view, abdominal sternite VIII
